# Molecular mechanism of antimicrobial activity of chlorhexidine against carbapenem-resistant *Acinetobacter baumannii*

**DOI:** 10.1371/journal.pone.0224107

**Published:** 2019-10-29

**Authors:** Deepika Biswas, Monalisa Tiwari, Vishvanath Tiwari

**Affiliations:** Department of Biochemistry, Central University of Rajasthan, Ajmer, India; North-Eastern Hill University, INDIA

## Abstract

*Acinetobacter baumannii* causes hospital-acquired infections, especially in those with impaired immune function. Biocides or disinfectants are widely used antibacterial agents used to eradicate the effect of *A*. *baumannii* on inanimate objects and health care environments. In the current study, the antimicrobial activity of chlorhexidine has been investigated against carbapenem-resistant (RS-307, RS-7434, RS-6694, and RS-122), and sensitive (ATCC-19606 and RS-10953) strains of *A*. *baumannii*. We have determined growth kinetics, antimicrobial susceptibility, ROS production, lipid peroxidation, cell viability using flow cytometry assay (FACS), and membrane integrity by scanning electron microscope (SEM). The effect of chlorhexidine on the bacterial membrane has also been investigated using Fourier transform infrared (FTIR) spectroscopy. The present study showed that 32μg/ml chlorhexidine treatment results in the decreased bacterial growth, CFU count and cell viability. The antibacterial activity of chlorhexidine is due to the elevated ROS production and higher lipid peroxidation. These biochemical changes result in the membrane damage and alteration in the membrane proteins, phospholipids, carbohydrates, nucleic acids as evident from the FTIR and SEM data. Therefore, chlorhexidine has the potential to be used in the hospital setups to remove the spread of *A*. *baumannii*.

## Introduction

*Acinetobacter baumannii* is non-fermentative, non-motile, strictly aerobic, non-fastidious, catalase positive, oxidase negative, and Gram-negative coccobacilli [1]. In last decades, this bacteria have turned into an important nosocomial pathogen from an opportunistic bacteria [2] due to its antibacterial resistance capability [3]. *A*. *baumannii* infections are associated with devastating outcomes in terms of mortality and morbidity and contribute to higher burden on the healthcare system. Skin and mucous membrane, wounds and burns, intravascular and urinary catheters, also urinary, gastrointestinal, and respiratory tracts are the sources of its infections [4]. Hospitals sources are the patient’s bed, mattresses, pillow, table, sinks, shower units, infusion pump together with suction and resuscitation equipment. *A*. *baumannii* has gained resistance to most of the antibiotics all over the world, which is the major challenge in the treatment. Hence, availability of effective antibiotics for *A*. *baumannii* treatment is restricted because of rapid increase in drug resistance [5].

Biocides are important components in the prevention of bacterial infections, commonly in intensive care units (ICUs) where there have been considerable reports on multidrug-resistant bacteria such as carbapenem-resistant *A*. *baumannii* [6]. Chlorhexidine, first synthesized in 1954 [7], is a biocide that is used as a skin disinfectant before surgery and to sterilize surgical instruments [8] and also against nosocomial infections in shared facilities [9].

Therefore, we have investigated into the molecular mechanism of chlorhexidine on the carbapenem resistant strain of *A*. *baumannii*. Various antimicrobial molecules showed ROS dependent membrane damage as an antibacterial mechanism hence we have explored this mechanism for chlorhexidine. In the present study, we have also investigated the antibacterial effect of chlorhexidine on membrane integrity and composition using flow cytometry, scanning electron microscopy (SEM) and fourier transform infrared spectroscopy (FTIR) respectively. These results will help in developing effective biocide to control the spread of *A*. *baumannii* infections, for hospital setups where antibiotics cannot be used.

## Material and methods

### Reagents

For the experiments, we have used various reagents, such as MacConkey broth, Luria bertani broth, Guanidine hydrochloride, Potassium Phosphate dibasic, Potassium phosphate monobasic, Sodium dodecyl sulfate, sodium hydroxide, Nitroblue tetrazolium (NBT), potassium hydroxide, 2,4-dinitrophenylhydrazine (DNPH), 2-Thiobarbituric acid (TBA), and Dimethyl sulfoxide, that were purchased from Himedia Laboratories Ltd., India. Chlorhexidine was purchased from Sigma Aldrich, Hydrochloric acid, sodium chloride and glycine were from Merck, India. Glacial acetic acid and glycerol from Qualigens, India. Ethyl acetate purchased from Fisher scientific.2-Thiobarbituric acid (0.8%) was prepared in 1M NaOH,2,4-dinitrophenylhydrazine (10mM) was prepared in 2N HCl. For PI staining, 100mM Tris (pH 7.4), 150mM NaCl, 1mM CaCl2, 0.5mM, MgCl_2_, and 0.1% Triton-X were prepared, 2.5% glutaraldehyde, and ethanol.

### Bacterial samples

The pre-collected bacterial strains of *A*. *baumannii* (RS307, RS6694, RS122, RS7434, RS10953 and ATCC-19606) were available in our laboratory at Central University of Rajasthan, Ajmer, India.

### Growth kinetics

Growth kinetics pattern of *A*. *baumannii* in the presence and absence of chlorhexidine was analyzed by UV-Vis Spectrophotometer at 605nm. For this, *A*. *baumannii* was cultured in LB media at 37°C under shaking conditions. Readings were taken at the regular time interval of 30min. When optical density reached at 0.6, the treatment of 16μg/ml, 32μg/ml and 64μg/ml of chlorhexidine was given. Differential growth curves were plotted to compare the bacterial growth, in the presence and absence of biocides. The concentration range was selected based on the previous published articles [10,11].

### Determination of bacterial cell viability using MTT assay

We have determined the bacterial cell viability using a modified MTT [3-(4, 5- Dimethyl-2-thiazolyl)-2,5-diphenyl-2H-tetrazolium bromide)] assay using published protocol [12–16]. The assay is based on conversion of yellow tetrazolium (MTT) into purple insoluble formazan crystals in the bacterial cytoplasm. This color change indicated the presence of live bacteria, whereas no color changes indicated the absence of live bacteria. In brief, firstly, 100μl cultures (OD of 0.2 at 605nm) of *A*. *baumannii* RS-307 and ATCC-19606 were transferred to 96 well-plate and treated with chlorhexidine (2μg/ml to 48μg/ml). After treatment, the multiwell plate was further incubated for 5 hours at 37°C. To determine the viable cells, 5μl of MTT solution (5mg/ml) was added to all wells and incubated for 1 hour in the dark at 37°C. After it 100μl of 100% DMSO was added to the suspension and incubated for 2hours at 37°C. Then, we measured the optical density of micro-plate at a wavelength of 570nm, and the percentage viability rate of wells was determined [17].

### Nitro blue tetrazolium assay

ROS have proposed to play critical roles in the bacterial response to lethal stress like antimicrobial peptide [18]. In oxidative stress conditions, ROS levels increases and causes significant damage to bacterial cell structures. Reports showed that chlorhexidine have role in ROS elevation to produce its antimicrobial effect [19–21]. Therefore, the reactive oxygen species (ROS) was measured by nitro blue tetrazolium assay (NBT) on a multi-well scanning spectrophotometer (ELISA reader) as per published protocol [22].

### Thiobarbituric acid assay

The thiobarbituric acid assay is most widely used to determine lipid peroxidation. During this process, the malondialdehyde (MDA) was formed by the decomposition of polyunsaturated fatty acids, which reacts with thiobarbituric acid. MDA (the secondary product) produce a pink-colored dimeric compound with thiobarbituric acid (TBA), which can be easily quantified using ELISA reader. We have quantified lipid peroxidation as per our published method [22].

### Colony forming assay

The numbers of viable cells were quantified by measuring colony forming units (CFUs) for both treated and untreated bacteria as per published protocol with some modification [23]. The culture was incubated at 37°C and monitored using UV spectrophotometer at 605nm until it reached the exponential phase (at OD 0.6). Control sample remained under growth condition while treatment was given with 32μg/ml chlorhexidine and further continuing cultivation for 3 hours. Pellet of 1 ml culture was diluted in 0.9% sodium chloride solution and prepared its dilutions from 10^1^ to 10^4^. From this, 10μl sample was taken from different reaction mixtures and spread on MacConkey agar plates using sterile glass rods. The plates were then incubated at 37°C for 16 hours (overnight). The number of viable cells after treatment with biocides were quantified and compared with untreated control to evaluate the antibacterial effect. The log reduction was calculated by log of ‘number of viable cells before treatment’ with ‘number of viable cells after treatment’.

### Flow cytometry analysis

Flow cytometry is a technique to enumerate the viable bacterial cell in the bacterial sample; here we have used treated and untreated bacterial samples. Propidium iodide is a nucleic acid dye that only leaks into the cells with ruptured cell membranes. Flow cytometry was prepared as per the method of Bankier et al. [24] with some modifications. To determine the viability status of bacterial cells, culture was grown until OD_600_ of 1 (10^9^ CFU). The 10μl primary culture is suspended into 1ml of Luria bertani broth (corresponding to 2 X 10^6^ to 1 X 10^7^ CFU/ml). These prepared bacterial cell suspensions (at log phase) were divided into three sets. One set was used for treatment with chlorhexidine, other for heat treatment (at 70°C for 30min as positive control) and third set was untreated control. All three samples were incubated for 3hours at 37°C. The incubated samples were centrifuged, pellet was treated with 3μM from PI (stock solution 1mg/ml, 1.5mM) and incubated for 30min in dark and the samples were subjected for detection of live and dead cells by flow cytometry (FACScan, BD Bioscience). Two non-fluorescent parameters determined the bacterial acquisition gate: forward scatter (FSC), X-axis and side scatter (SSC), Y-axis, and one fluorescent parameter (red fluorescence) from PI. FSC and SSC channels help to eliminate background noise. The threshold was also set at the FSC signals of control.

### Fourier transform infrared spectroscopy (FTIR)

Recently, FTIR being increasingly used to investigate the mode of action of bactericidal compounds and to determine changes in bacterial cells in response to different stresses [25]. *A*. *baumannii* strains were grown in LB broth till OD reaches 0.6 and divided into two sets. One set was treated with chlorhexidine while other was used as control. The bacterial cultures in different conditions were centrifuged at 13000RPM for 15min and the supernatant was discarded. Bacterial pellets were washed (twice) with phosphate buffer solution by centrifugation at 13000RPM for 3 minutes. All FTIR spectra were recorded in the transmission mode, by the potassium bromide (KBr) disc method. The FTIR spectra were recorded with the PerkinElmer Spectrum Bx FTIR system spectrophotometer Version 10.4 with deuterated triglycine sulfate (DTGS) detector elements. FTIR data was obtained in the wavelength range 4000–400 cm^-1^, 32 scans at a pixel resolution of 8 cm^-1^ and spectral resolution 4 cm^-1^. The pellets, with potassium bromide (KBr) were grinded in a mortar and pestle. The obtained background from KBr disc was automatically subtracted from the prepared KBr and sample disc spectra.

### Field emission scanning electron microscopy (FESEM)

The field emission scanning electron microscope (FESEM) is a useful technique to study the morphological features of bacteria [26]. The ultrastructural changes in bacterial morphology caused by disinfectant were examined by field emission scanning electron microscopy (FESEM). The appropriate growth conditioned bacterial suspension, while was grown in LB broth until the OD reached at 0.6, was divided into two sets. One set was treated with 32μg/ml chlorhexidine while other was kept as untreated and incubated at 37°C until the OD of control reached at 1.0. 4ml bacterial cells (treated and untreated) were centrifuged at 13000 rpm for 30 minutes at 4 °C. The obtained pellets were washed with PBS to remove the remaining medium before the pellets were fixed with glutaraldehyde. A solution (100 μl) of 2.5% glutaraldehyde in PBS (pH 7) was added to the pellet and incubated at 4 °C for 2.5 hours. The pellet was washed with PBS and fixation step was repeated. After fixation, cells were washed 4 times for 30 minutes with PBS at 4 °C. Finally, 20μl PBS was added to the pellet, mixed and one drop of this suspension was placed on the glass cover slip. The smear was allowed to dry and then dehydrated with ethanol gradients (35%, 50%, 75%, 95% and 100%) followed by air dry. The fixed bacterial smears were then coated with a layer of gold (Quorum) and the conductive samples were examined with SEM (Nova NanoSEM 450).

### Statistical methods

The data was analyzed for standard deviation based on the entire data using the STDEV function in Microsoft excels. The results were expressed as the mean ± the standard error of the mean (n = 3). For the statistical method, we have used graph pad prism and performed the *t-test* to compare the findings.

## Results

In the present study, the experiments were designed to identify the biochemical alterations of biocide-treated multidrug resistance strains of *A*. *baumannii*. The focus of this study is to have a comparative difference in the *A*. *baumannii* after biocide treatment using biochemical and FTIR based signatures, which might indicate the molecular mechanism of biocide. In the present study, we have used carbapenem-resistant strains of *A*. *baumannii* which were found to be highly resistant (RS307, RS6694, and RS122), intermediate carbapenem-resistant (RS7434) and sensitive (RS-10953 and ATCC-19606) strains.

### Growth kinetics showed the inhibitory effect of chlorhexidine

**Fig 1:**
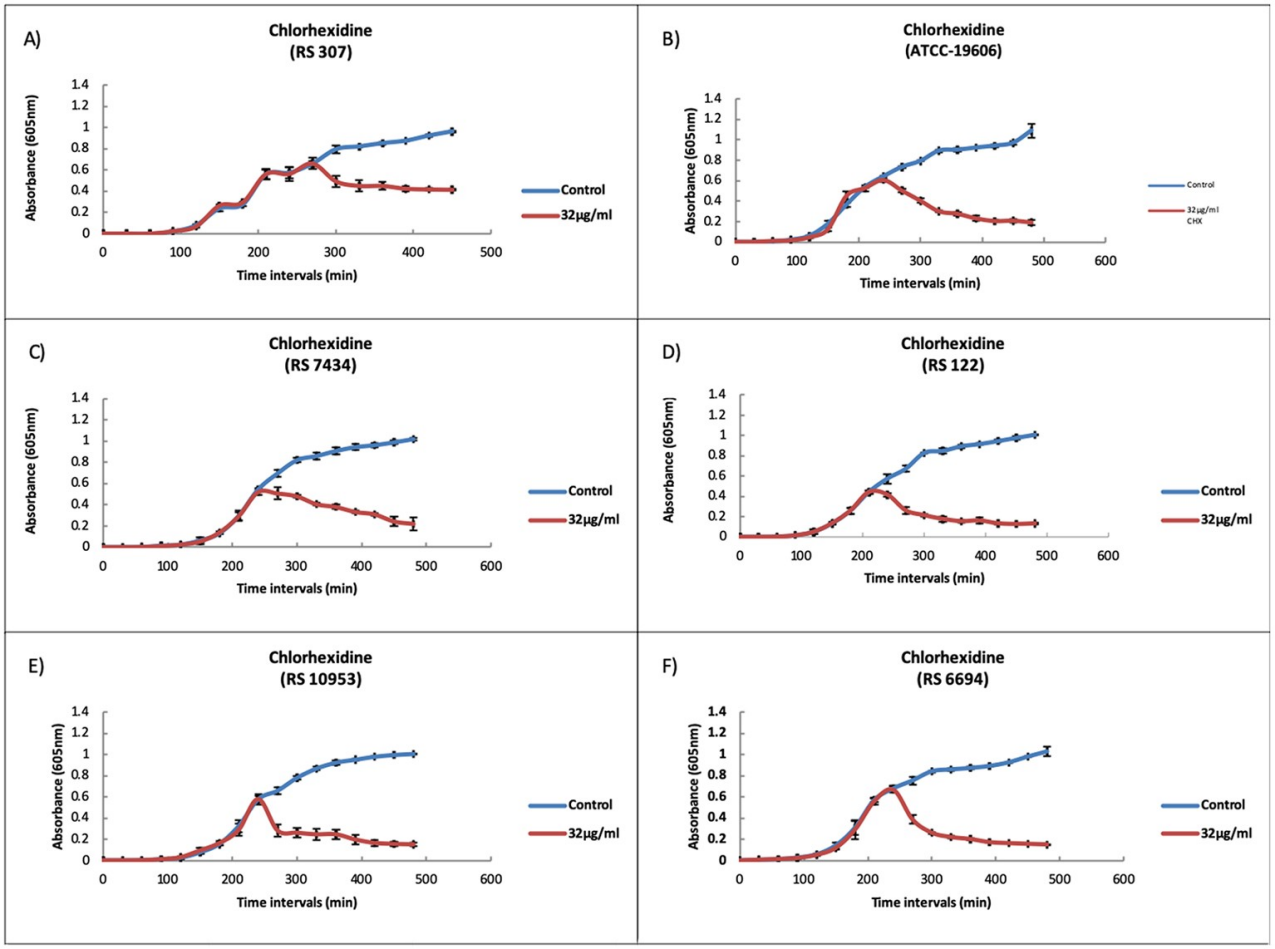
Growth kinetics of *A*. *baumannii* strains were determined in the presence and absence of effective concentration of chlorhexidine that measured by UV-Vis spectrophotometer at 605 nm. Data expressed is Mean± SEM of 3 values (n = 3) (Control: Average of Control; 32μg/ml Chlorhexidine). Results of 16μg/ml and 64μg/ml chlorhexidine treated samples are provided as S1 Fig.

The result of growth analysis of ATCC19606 and RS307 showed that 32μg/ml and 64μg/ml showed a similar effect, therefore 32μg/ml were used in further study. The result showed that chlorhexidine has significant inhibitory effect on the strains RS307 (p-value 0.0019), ATCC19606 (p-value 0.0023), RS6694 (p-value 0.002), RS122 (p-value 0.0015), RS7434 (p-value 0.0063), and RS10953 (p-value 0.0025) (Fig 1). This result showed that the 32μg/ml chlorhexidine have inhibitory effect on different strains of *A*. *baumannii*.

### Microtiter plate analysis also showed the inhibitory effect of chlorhexidine

*A*. *baumannii* strains RS-307 and ATCC-19606 were treated with various concentration of chlorhexidine (2μg/ml-48μg/ml). The different concentrations of disinfectant produce a different inhibitory effect such as >50% inhibitory effect on RS307 at 2μg/ml (25.64% cell viability) and ATCC-19606 (12 μg/ml, 28.97% cell viability). Microtiter experiment also confirms the inhibitory effect of chlorhexidine on *A*. *baumannii*.

### Chlorhexidine treatment decreases CFU by *Acinetobacter baumannii*

**Fig 2:**
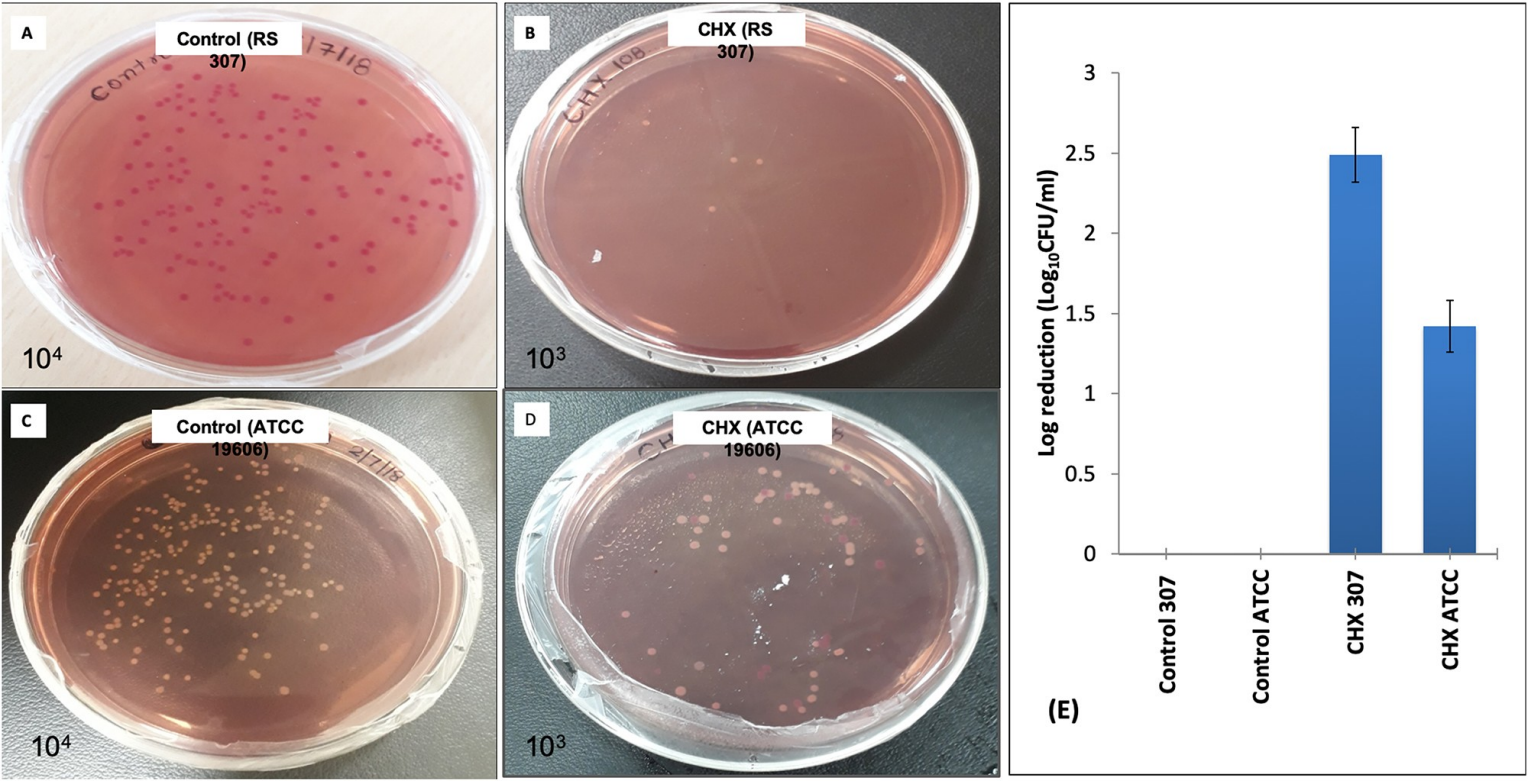
Representative culture plate of colony forming unit count of *A*. *baumannii* strains untreated and treated with 32 μg/ml Chlorhexidine: (A) Control untreated RS-307; (B) Chlorhexidine treated RS-307; (C) Control untreated ATCC-19606; (D) Chlorhexidine treated ATCC-19606; (E) Log reduction plot for chlorhexidine treatment. Plates showing few or no bacterial growth after treatment. Experiment performed in triplicates, shown the final result observed.

Colony forming unit (CFU) measurements indicate the antibacterial effect of chlorhexidine against the strains of *A*. *baumannii*. The viability of both bacterial strains was reduced in the presence of 32 μg/ml chlorhexidine concentration. The bacterial colonies after 10^3^ dilutions of RS307, clearly showed that after treatment, there is a decrease in the number of CFU in the treated sample as compared to control (untreated) (Fig 2A and 2B). Similarity, bacterial colonies after 10^3^ dilutions of ATCC-19606 showed decreases in CFU after treatment (Fig 2C and 2D). Growth of the bacterial cells was less than 5% after the incubation at 37° C for 16 hours. CFU of RS307 at 10^3^ dilutions is 1940 x 10^4^ while for ATCC, it is 1270 x10^4^. The treatment with chlorhexidine results in the decrease of CFU for RS-307 (33x10^4^) and for ATCC-19606 (340 x10^4^). The treatment of chlorhexidine result in 2.49 and 1.42 log reduction of RS307 and ATCC-19606 strain of *A*. *baumannii* respectively as compared to the control (Fig 2E).

### Enhancement of ROS generation in *A*. *baumannii* by chlorhexidine

**Fig 3:**
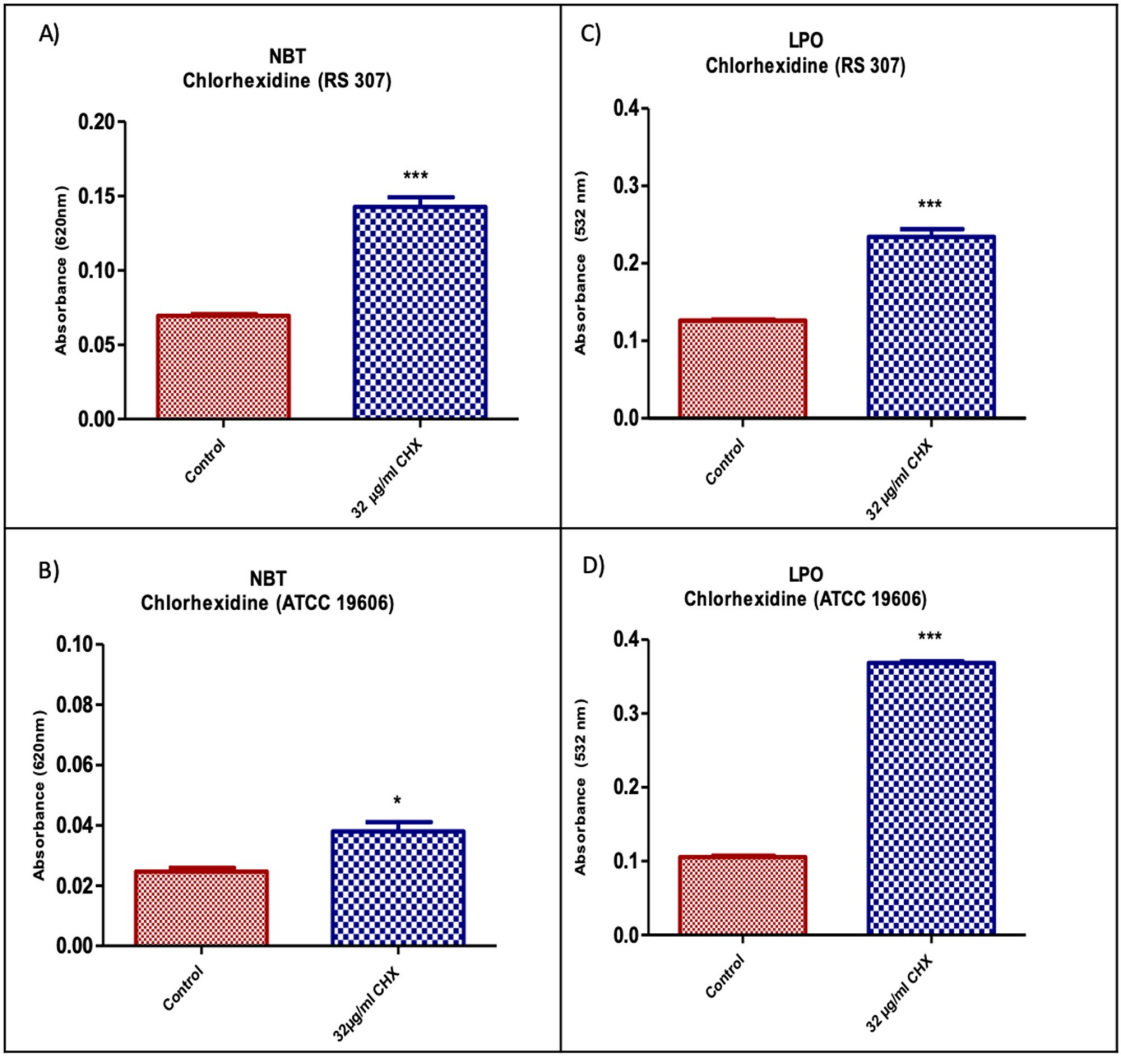
(A and B) Increase in the generation of total ROS using NBT as a substrate in treated *A*. *baumannii* strains RS307 and ATCC 19606 with chlorhexidine as compared to the control. (C and D) Absorbance values of *A*. *baumannii* strains RS307 and ATCC 19606 after treated with chlorhexidine as compared to the untreated control sample. The data expressed are mean ± SEM of at least three values (n = 3). Values were measured via the ELISA reader. ELISA, enzyme ‐linked immunosorbent assay; NBT, nitroblue tetrazolium.

Chlorhexidine (32μg/ml) treated *A*. *baumannii* strains (RS307 and ATCC-19606) shows the significant elevated ROS generation in the treated bacterial samples as compared to the untreated bacterial samples (Fig 3A and 3B). Furthermore, 32μg/ml chlorhexidine treatment resulted in two folds ROS generation in the resistant strain.

### Elevated lipid peroxidation after chlorhexidine treatment

Lipid peroxidation results showed that higher MDA production has seen after treatment with 32μg/ml chlorhexidine in the strain RS-307 as well as in ATCC-19606 as compared to untreated. This suggests that 32μg/ml chlorhexidine is significantly effective in lipid peroxidation of *A*. *baumannii* (Fig 3C and 3D).

### Flow cytometry also confirmed the bacterial membrane disruption after chlorhexidine

**Fig 4:**
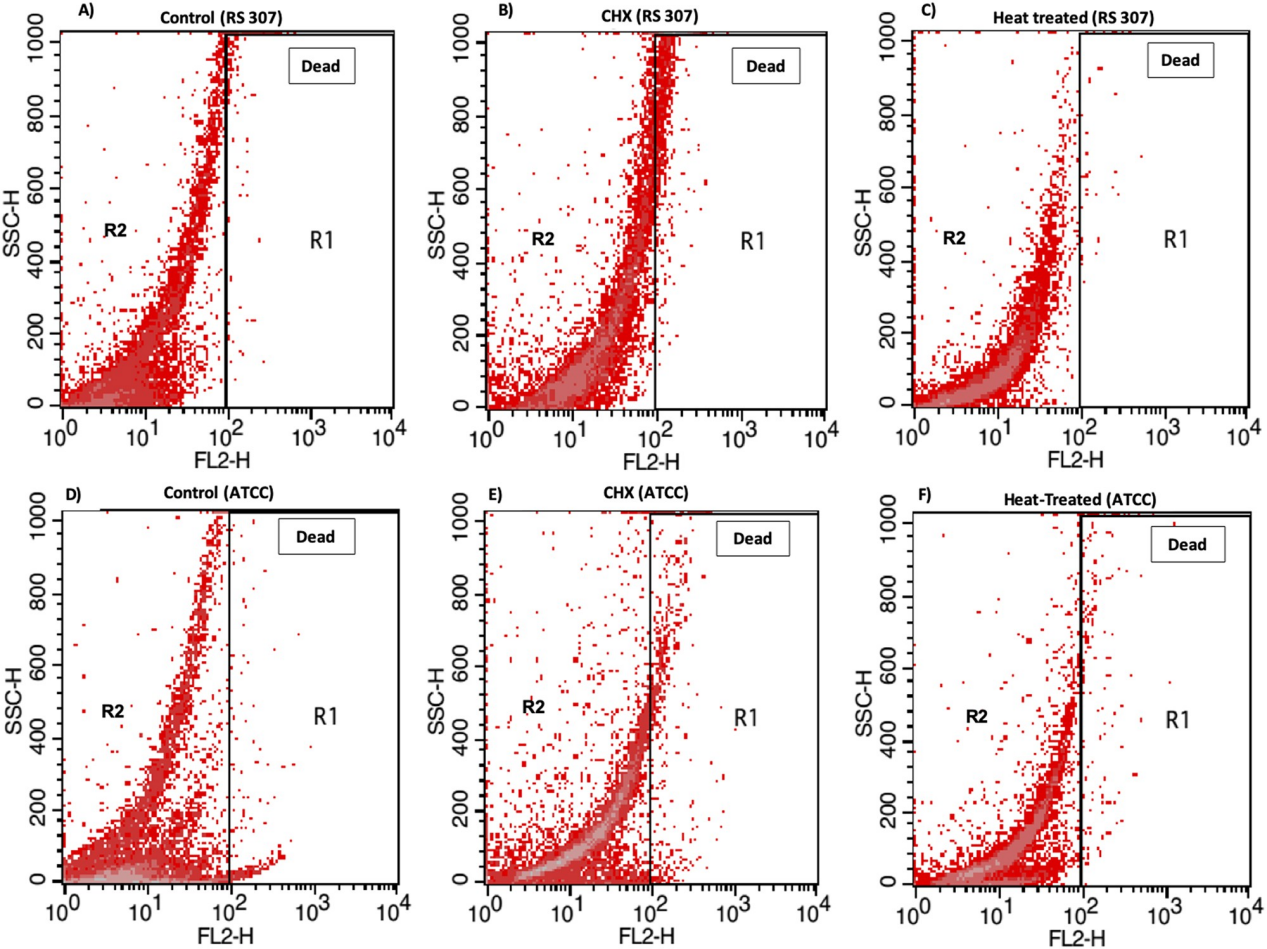
Flow cytometry dot plots of *A*. *baumannii* strains (RS 307 and ATCC): (A) Control (RS 307) (B) CHX treated (RS 307) (C) Heat-treated (RS 307)) (D) Control (ATCC) (E) CHX treated (ATCC) (F) Heat-treated (ATCC). Histogram graphs of flow cytometry are provided as S2 Fig.

Flow cytometric analysis of *A*. *baumannii* viability control showed that the bacteria were more viable as compared to the treated bacteria (Fig 4). The heat-treated dead cells showed less viable cells as compared to control. Chlorhexidine treatment showed more inhibitory effect as compared to control and heat-treated cells (PI-positive cells). Cells coming in the range of R2 zone of autofluorescence from unstained cells are set within the first log decade (10^0^ to 10^1^). The result showed that there is a difference in the Geo-mean of untreated RS307 (6.19), heat treated RS307 (6.66), chlorhexidine-treated RS307 (23.46). Similarly, Geo-mean of ATCC-19606 strain also showed a difference in untreated (9.11), heat-treated ATCC-19606 (9.61), chlorhexidine-treated ATCC 19606 (14.94). The events count for untreated, chlorhexidine-treated and heat treated was found to be 247, 1904 and 202 for RS307 while 300, 515 and 135 events for ATCC-19606 respectively. These results state that there are more dead cells observed in the treated samples as compared to the untreated samples. Flow cytometry was also used to monitor the cell membrane integrity or disruption, and a positive PI cells in flow cytometry showed membrane disruption [27,28]. The significant difference shown between control, and treated bacterial cells suggests changes in *A*. *baumannii* cell membrane integrity after chlorhexidine treatment.

### FTIR signature in the control and resistant strain of *A*. *baumannii*

**Fig 5:**
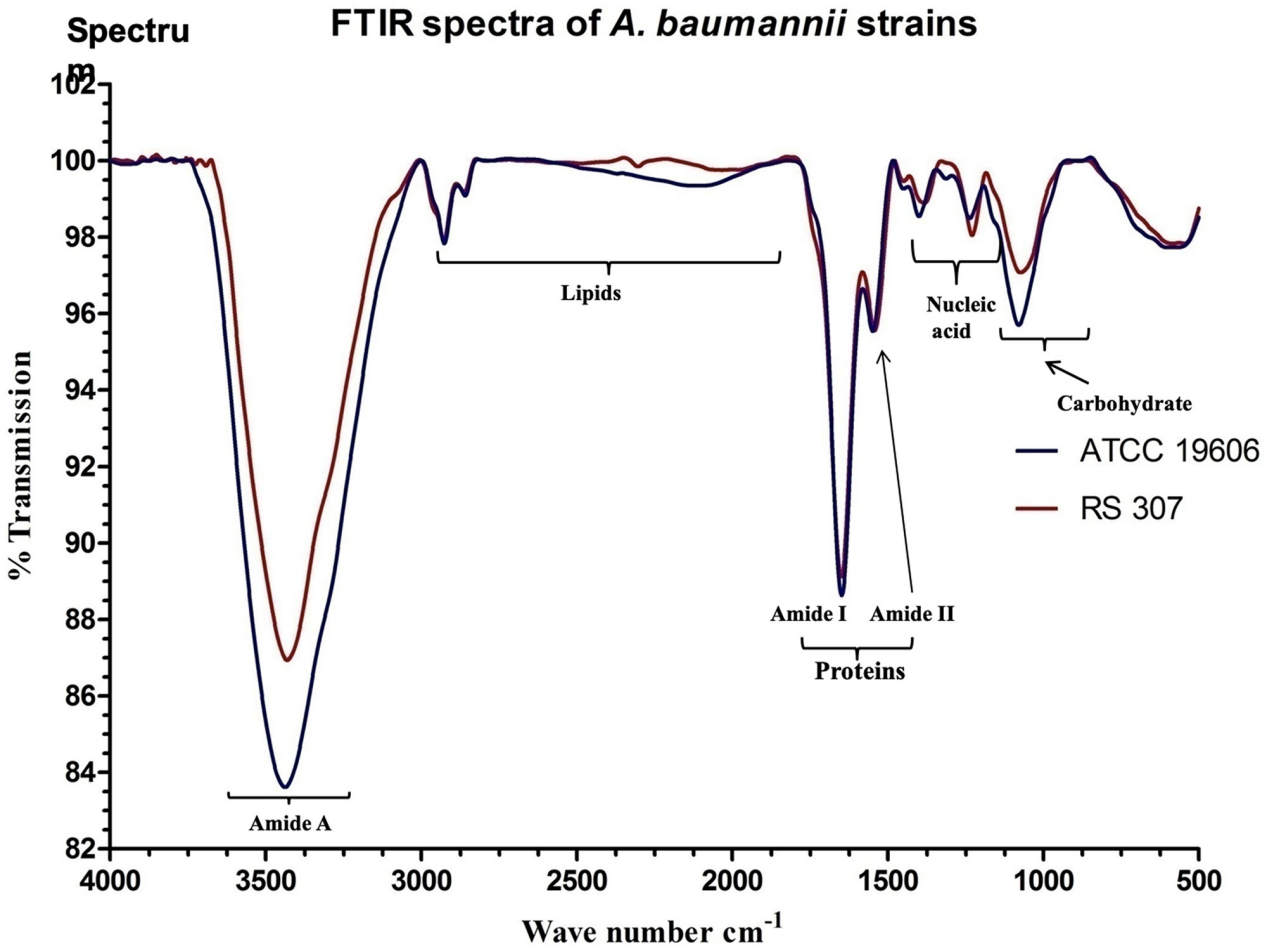
Comparative FTIR spectra (500–4000 cm^-1^) of *A*. *baumannii* strains (ATCC 19606 and RS 307).

FTIR spectra of the control ATCC and resistant strain RS307, showed biomolecules characteristics IR bands: lipids (3000–2800 cm^-1^), protein/amides I and II (1700–1500 cm-1), phospholipids/DNA/RNA (1500–1185 cm^-1^), polysaccharides (1185–900 cm^-1^) and fingerprint region (900–600 cm^-1^) [29]. The phospholipids/DNA/RNA and carbohydrate region are previously used to discriminate different clones of *A*. *baumannii* [30]. FTIR spectra of both the strains are very similar, but some differences are also observed in both the spectra (Fig 5). The comparative spectra showed the minor shift in spectra from around 1400 cm^-1^ in ATCC-19606 to 1382cm^-1^ in RS-307, this showed the difference in the fatty acid chains of phospholipids and amino acid side chain [31]. Similarly, there is a minor shift in FTIR spectra form around 1240cm^-1^ in ATCC-196906 to 1230 cm^-1^ in RS-307 which showed changes in phospholipids and some nucleic acids of two strains [32]. Comparative FTIR result around 600nm also showed that there is a minor change in the carbohydrate composition of these two strains. The resistant strain also has more proteins (Amide A band at 3430cm^-1^) and phospholipid (1080cm^-1^) as compare to sensitive strain. These changes in the carbohydrate and phospholipid may be associated with the higher drug tolerance of the resistant strain.

### FTIR spectra confirm the alteration in membrane proteins, phospholipids, nucleic acids and carbohydrate by chlorhexidine

**Fig 6:**
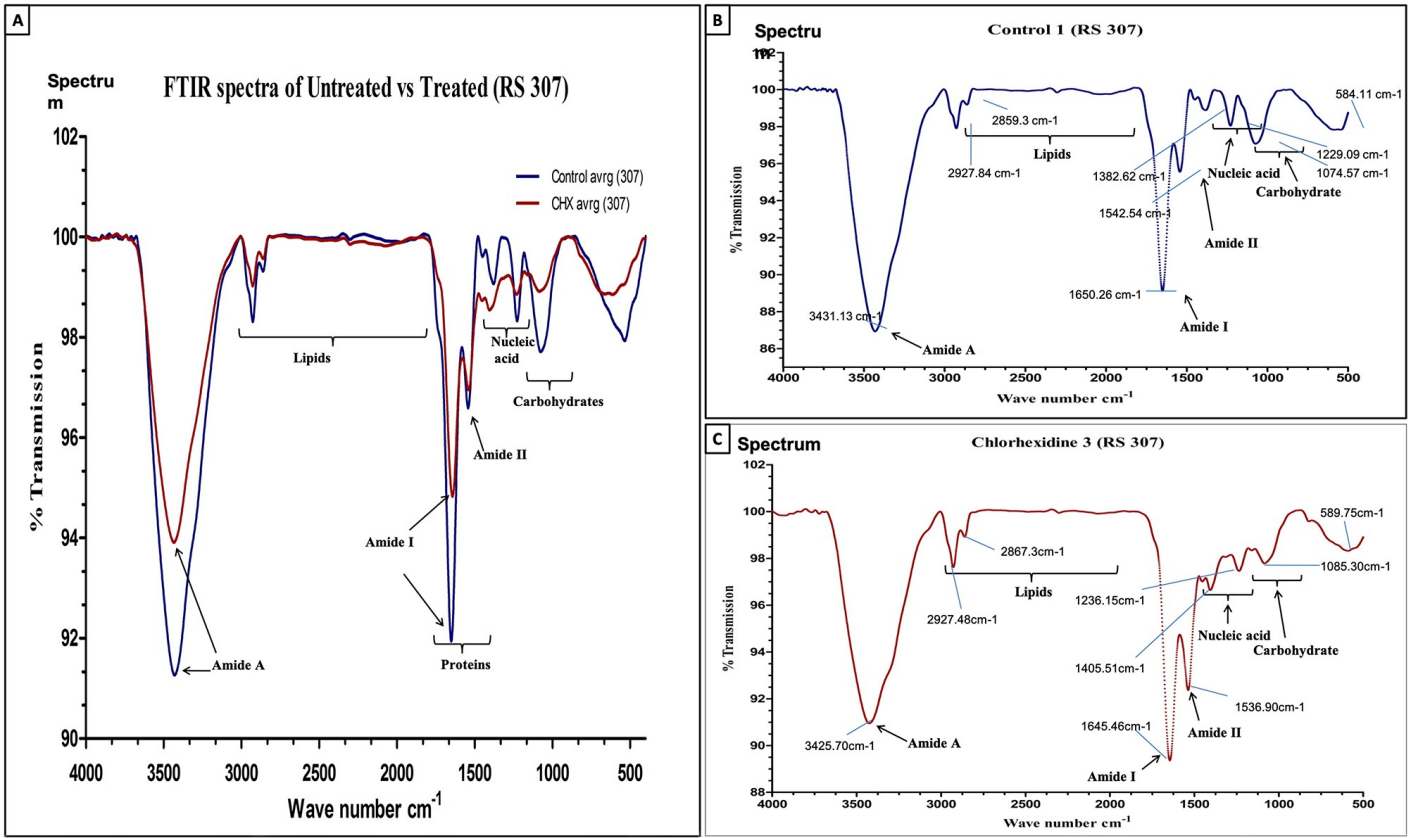
FTIR spectroscopy-based spectra (500–4000 cm^-1^) of (A) Combined spectra of Control and 32 μg/ml CHX treated *A*. *baumannii* (RS 307). (B) Control. and (C) 32 μg/ml CHX Treated, in transmission mode. The experiment was performed in triplicates. Results of two sets are provided as S3 Fig.

**Fig 7:**
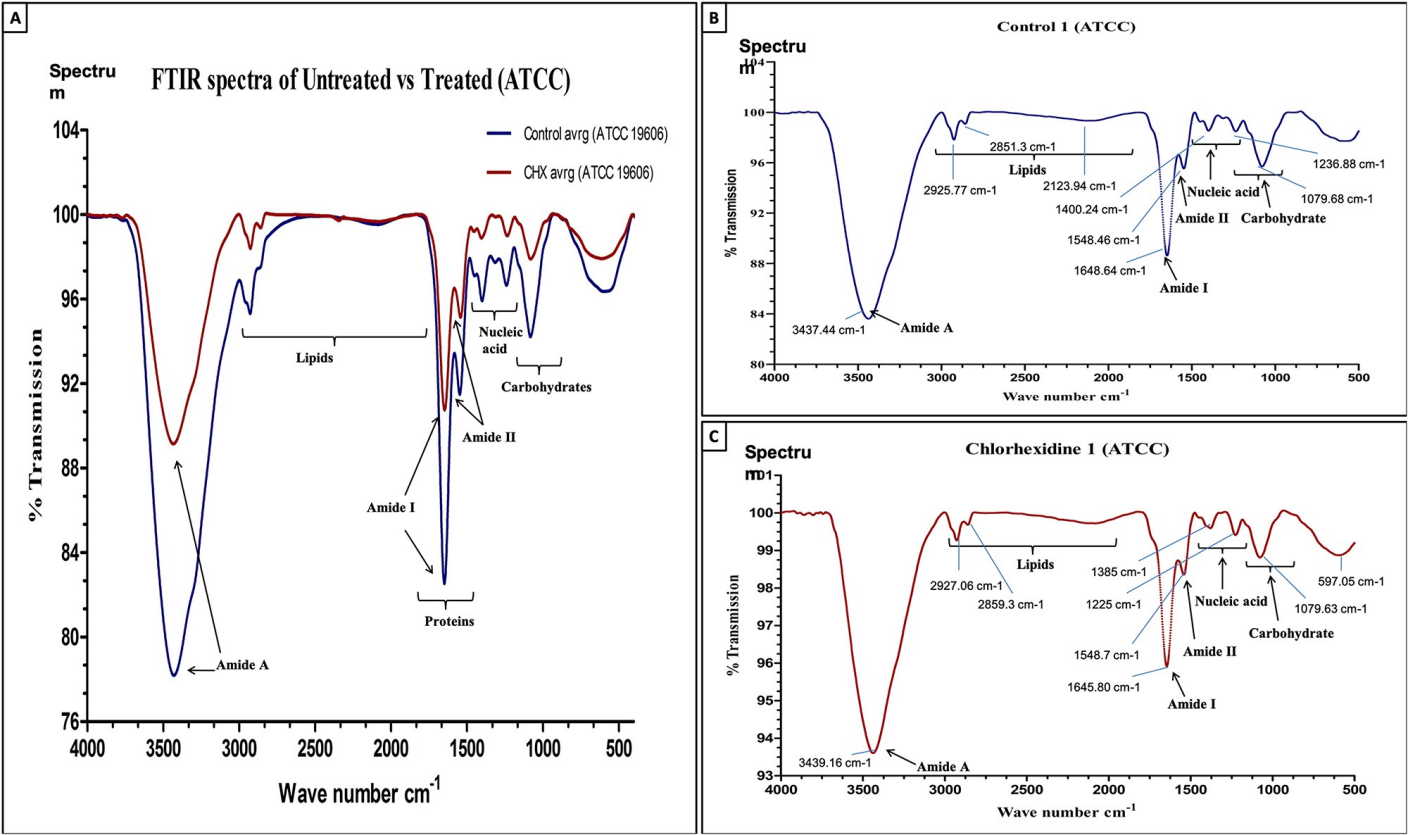
FTIR spectroscopy-based spectra (500–4000 cm^-1^) of (A) Combined spectra of Control and 32 μg/ml CHX treated *A*. *baumannii* (ATCC). (B) Control. and (C) 32 μg/ml CHX Treated, in transmission mode. The experiment was performed in triplicates. Results of two sets are provided as S4 Fig.

To investigate the effect of chlorhexidine on the *Acinetobacter baumannii*, we have recorded the FTIR spectra for chlorhexidine treated and untreated strains of *A*. *baumannii* (ATCC-19606 and RS-307). The experiments were performed in triplicates and have obtained individual spectra of each sample. This experiment was performed using the whole bacterial culture, and we found out a significant spectral difference in the untreated and treated samples, associated with absorbance of macromolecules such as proteins, nucleic acids, carbohydrates and phospholipids. Changes in the shape and frequencies are noticeable (Figs 6 and 7). It was observed that after chlorhexidine treatment, there is a change in the transmittance or absorbance of IR signal for nucleic acids, carbohydrates, protein and phospholipids in the treated sample as compared to the untreated sample. Results implicate the significant shift in the IR spectra for stretching modes of phosphates of phospholipids (1240cm^-1^), bending of CH_2_ backbone of phospholipids (1460cm^-1^), rocking of CH_2_ (720cm^-1^) deformation of asymmetric CH_3_ (1405cm^-1^), phospholipids (1191cm^-1^) and lipids esters (1740cm^-1^) after treatment with chlorhexidine. These finding suggests damage of phospholipid and degradation of lipid esters of the membrane. Chlorhexidine treatment also changes the protein structures that are evident from the amide I and amide II bands. It was observed that chlorhexidine treatment resulted into shift in the alpha-helix in range of 1648–1660cm^-1^ (1652cm^-1^ in amide I and 1548cm^-1^ in amide II), shift in beta-sheet (1630cm^-1^ for anti-parallel beta-sheet and 1640cm^-1^ for parallel beta-sheet)in the range of 1620–1640cm^-1^, main component) and 1670–1695cm^-1^, second frequency, and turn (1680cm^-1^)in the range of 1620–1640cm^-1^, main component and 1650–1695cm^-1^, second frequency. This suggests that the *Acinetobacter* membrane disruption and protein degradation, or denaturation is the target of the chlorhexidine.

Similarly, further analysis of chlorhexidine on nucleic acid of ATCC-19606 showed that there is a shift in the FTIR signature peak of PO_2_^-^ from 1229cm-1 in B-DNA spectrum to 1239cm^-1^ in the A-DNA spectrum [33]. This showed that chlorhexidine also causes conformational changes in the DNA to inactivate the *Acinetobacter baumannii*. In resistant strain RS307, amide I peak of untreated sample is located at 1650 cm^-1^ (%T 89.122502), while in case of chlorhexidine treated sample, the peak observed at around 1634.78 cm^-1^ (% T 98.898112). This showed that chlorhexidine also induce some alpha-helix (1650cm^-1^) to beta sheet (1635cm^-1^) transition in protein structure [32]. These two observations need further analysis before confirmation.

### Scanning electron microscopy confirms membrane disruption by chlorhexidine

**Fig 8:**
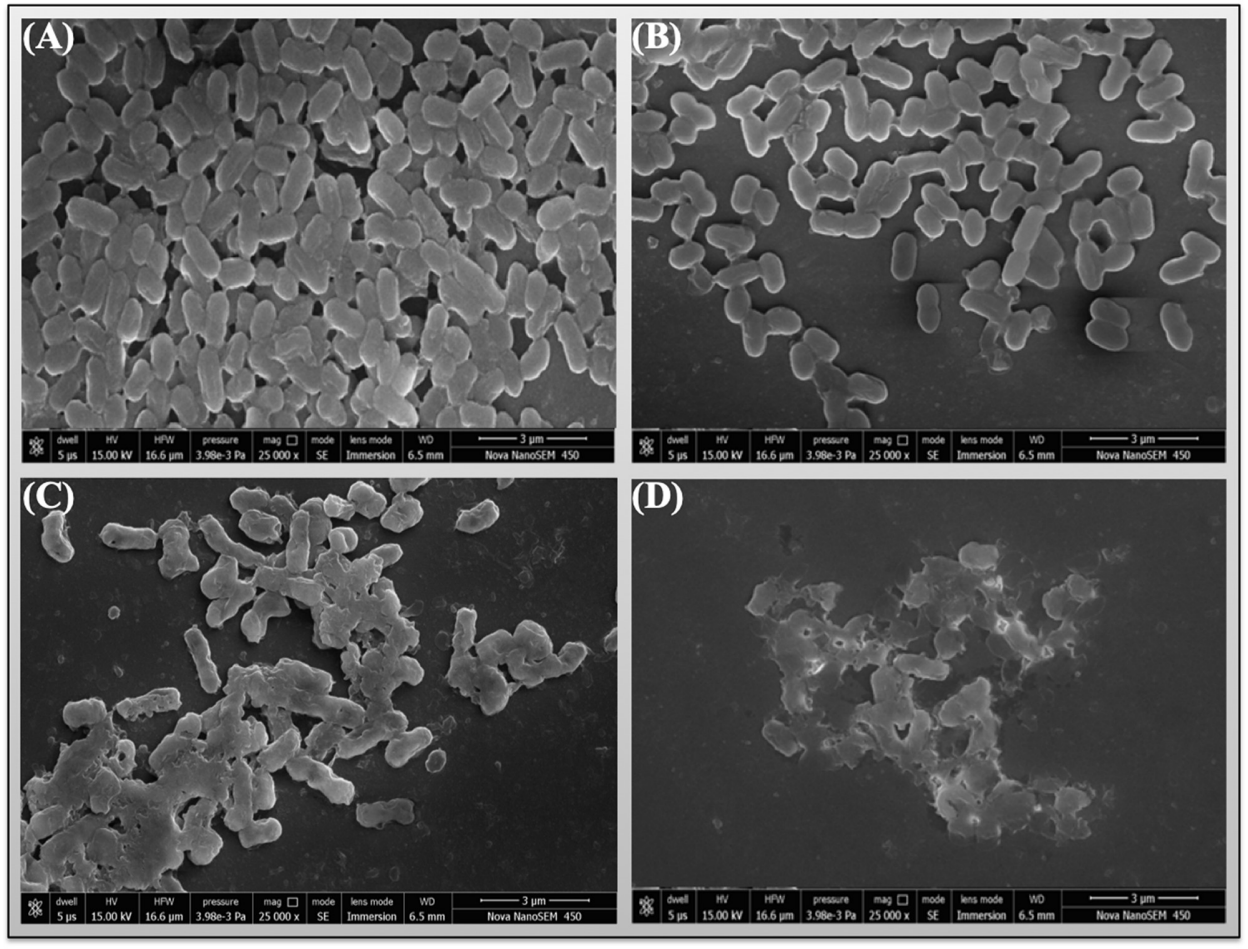
Scanning electron microscopy (SEM) images of *A*. *baumannii* cells after chlorhexidine treatment. SEM image of untreated RS-307 strain (A); untreated ATCC-19606 (B); RS-307 treated with chlorhexidine (C); ATCC-19606 treated with chlorhexidine (D). (magnification: 25000 X; SE mode; Bars: 3μm).

FESEM analysis of *A*. *baumannii* (strains RS-307 and ATCC-19606) bacterial cells showed observable change in cellular morphology post exposure to chlorhexidine (Fig 8C and 8D) as compared to control (Fig 8A and 8B). The figure also showed that the treatment of chlorhexidine resulted into the loss of cellular integrity and membrane rupture, which further supports the results from other techniques like FTIR, flow cytometry etc.

## Discussion

Various studies have investigated that bacteria can persist in the hospital environment from several days to a few months. Due of this, the hospital environment becomes a reservoir for hospital-associated bacteria and contributes to their transmission. There is an increasing evidence that effective biocide of the hospital environment plays an essential role in controlling the transfer of multi-resistant bacteria. *Acinetobacter* have developed resistance mechanism to most of the antibiotics [5,34–37] and different approaches [38–41] have been investigated to control this pathogen. Studies have been done to demonstrate the antimicrobial activity of biocides. Still, the mechanism behind the antibacterial activity is a matter of intensive research [42]. Biocides are the chemical agents inactivate virtually all recognized pathogenic bacteria. *A*. *baumannii* is a critical nosocomial pathogen with resistance to various antimicrobial agent [43]. It is an opportunistic and multidrug-resistant pathogen [44]. When they build up their biological community in a hospital, it is tough to get them out, since they thrive in respirator tubing, as well as on inanimate surfaces, for example, tabletops and bed guardrails [45]. Therefore, biocides play a very important role to irradiate it. It is generally accepted that biocides have multiple target sites within the bacterial cell, and overall damage to these target sites results in the bactericidal effect; but insusceptibility mechanism of biocides may be similar, whereas not necessarily identical [46]. Chlorhexidine has been used to eradicate the nosocomial infections; present on the hospital environment or inanimate objects [47]. In a recent study, the effect of chlorhexidine bathing as compared to routine bathing has been tested in multi-drug resistant organisms [48]. Increased usage of disinfectants has not been matched by an increase in surveillance [42]. This might be because of the reasons for the lack of surveillance is the lack of both standardized testing methods and definitions for reduced susceptibility.

Previously it was shown that 32μg/ml chlorhexidine is effective biocide against MRSA [11]; therefore we have screened different concentrations of chlorhexidine (16μg/ml, 32μg/ml and 64μg/ml). The results state that 32μg/ml and 64μg/ml chlorhexidine showed similar antibacterial activity; hence, we had selected 32μg/ml for further analysis. Our result demonstrated that chlorhexidine effectively reduces the number of multi-drug resistance strains of *A*. *baumannii*. This was confirmed by CFU and growth kinetics assay. To investigate the molecular mechanism, we have also monitored the involvement of reactive oxygen species by nitro blue tetrazolium assay and thiobarbituric acid assay. Higher production of ROS may damage varieties of cellular macromolecules, for example, RNA, DNA, protein and lipids etc which leads to loss of function, an increased rate of mutagenesis and ultimately bacterial cell death [49]. Result of NBT assay has shown increases ROS in treated bacteria with 32μg/ml chlorhexidine in both the strains, also increased in lipid peroxidation. Colony forming unit was found to be less in the *Acinetobacter baumannii* treated with chlorhexidine as compared to untreated.

We performed flow cytometry for determination of the dead cells present in the treated and untreated bacterial culture. For the experiment, we have used Propidium iodide (PI), a red-fluorescent nuclear staining dye that is used to analyze the dead cells in a population [50]. The result of the flow cytometry assay in the strain RS 307 and ATCC 19606 demonstrated that the control sample appeared with the less dead cell as compared to the chlorhexidine treated and heat-treated cells.

FTIR spectroscopic analysis has observed as a label-free characterization [51]. Each bacterial species has a complex cell wall composition, which gives a unique FTIR metabolic fingerprint. This distinctiveness is because of the stretching and bending vibrations of molecular bonds or functional groups present in its biomolecules, such as proteins, nucleic acid, lipids, sugars, and lipopolysaccharides within the cell wall. The molecular compositions can alter from species to species and at the level of strain. Therefore, all the bacteria will have their unique and characteristic spectrum, and single microorganisms could be identified from an FTIR spectrum. It was measured for intact bacterial cells that are complex, and the peaks are broad because of superposition of contributions from all the biomolecules present in the cells of bacteria. The results showed similarities in the FTIR signature of ATCC-19606 and resistant strain RS-307 but some difference in the phospholipids and carbohydrate reason. The treatment of chlorhexidine to the ATCC-19606 and RS-307 result in different FTIR pattern but both FTIR spectra confirms the damage in the membrane, alteration in phospholipids, protein and nucleic acids. SEM results also confirmed the membrane disruption by chlorhexidine. The overall results indicate that chlorhexidine is an effective disinfectant against the *A*. *baumannii* strains and leads to the membrane damage to exert its antibacterial effects.

## Conclusion

The present result concludes that the growth kinetics graph and CFU counting after 32μg/ml chlorhexidine treatment result in bacterial cell death. Based on flow cytometry, FTIR and SEM analysis, the probable mechanism of action of chlorhexidine involves a ROS dependent membrane damage and alterations in membrane compositions. The membrane damage is a result of oxidative stress and lipid peroxidation in the bacterial cell. FTIR signature shows similarly in the *Acinetobacter baumannii* ATCC-19606 and RS-307 while the FTIR spectra also pointed out some differences after chlorhexidine treatment. The results highlighted that the FTIR spectra change attributed mainly to phospholipids and proteins as well as minor changes in the nucleic acids. They also enlighten the use of FTIR to analyze the molecular mechanism of disinfectants against *Acinetobacter baumannii*. Further studies are required to determine the precise molecular mechanism of disinfectants. Further, a dose-dependent study may also be needed to enhance the outcomes of this study.

## Supporting information

S1 Fig Growth kinetics of *A*. *baumannii* strains in the presence and absence of different concentrations of chlorhexidine. (TIFF)

S2 Fig Histogram graphs showing the results of cell counts in flow cytometry of untreated and treated samples of *A*. *baumannii*. (TIFF)

S3 Fig FTIR spectroscopy-based spectra (500–4000 cm^-1^) of two sets of untreated and chlorhexidine treated sample of RS307. (TIFF)

S4 Fig FTIR spectroscopy-based spectra (500–4000 cm^-1^) of two sets of untreated and chlorhexidine treated samples of ATCC 19606. (TIFF)
