# Supplementary figures and images for: Molecular mechanism of antimicrobial activity of chlorhexidine against carbapenem-resistant Acinetobacter baumannii
Source: PLoS One. 2019 Oct 29;14(10):e0224107. doi: 10.1371/journal.pone.0224107 (PMC6818764; doi:10.1371/journal.pone.0224107)

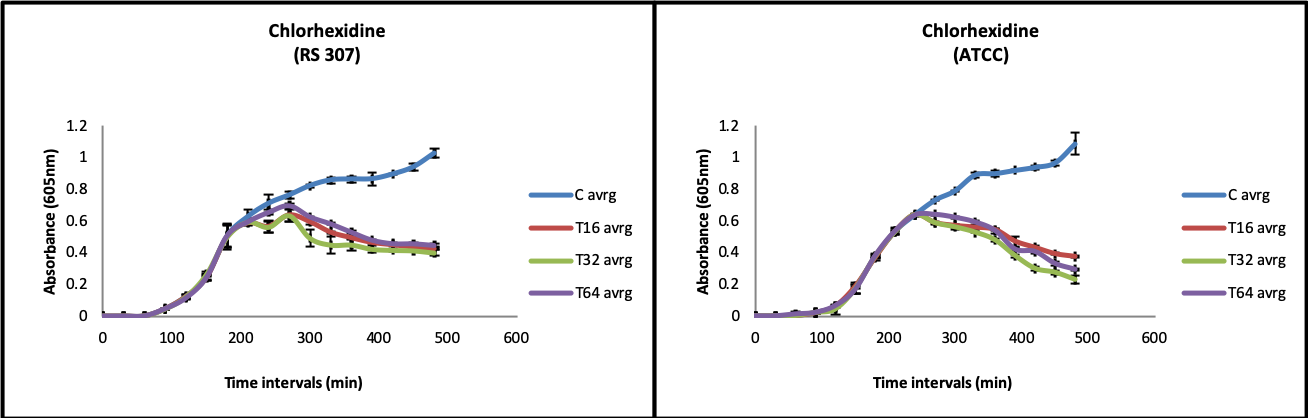

Supplement: S1 Fig — (TIFF) [file pone.0224107.s001.tiff]

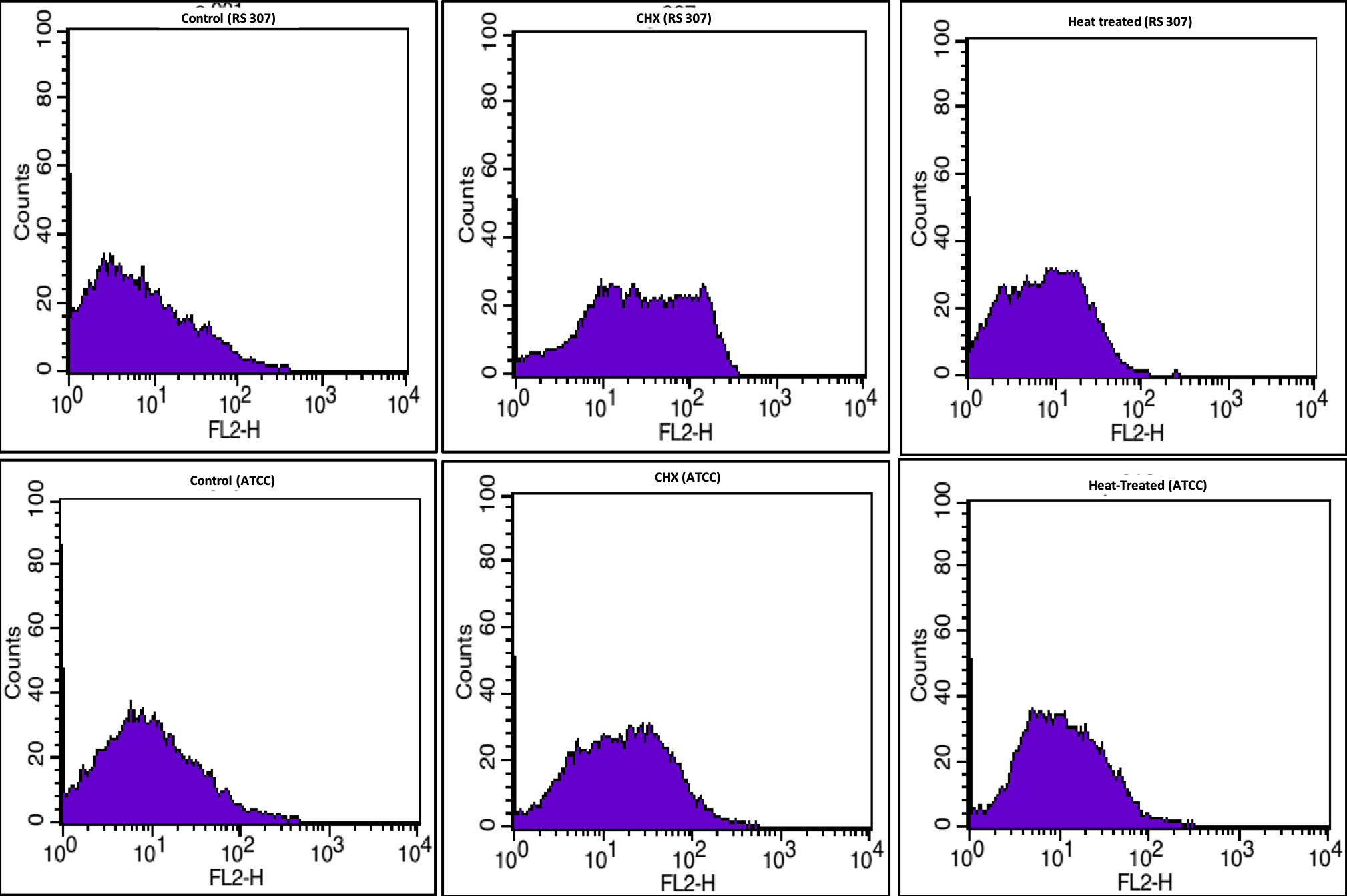

Supplement: S2 Fig — (TIFF) [file pone.0224107.s002.tiff]

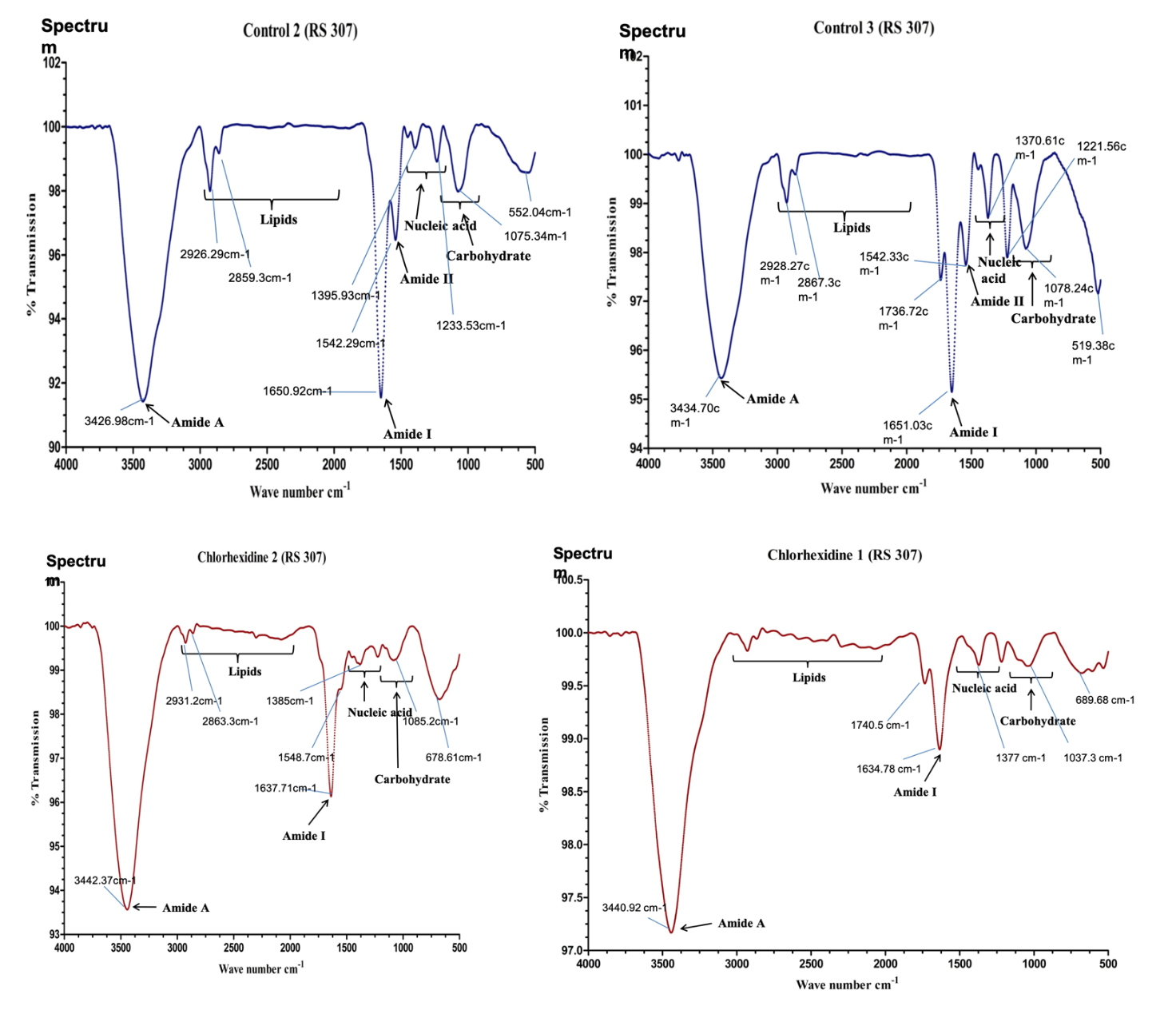

Supplement: S3 Fig — (TIFF) [file pone.0224107.s003.tiff]

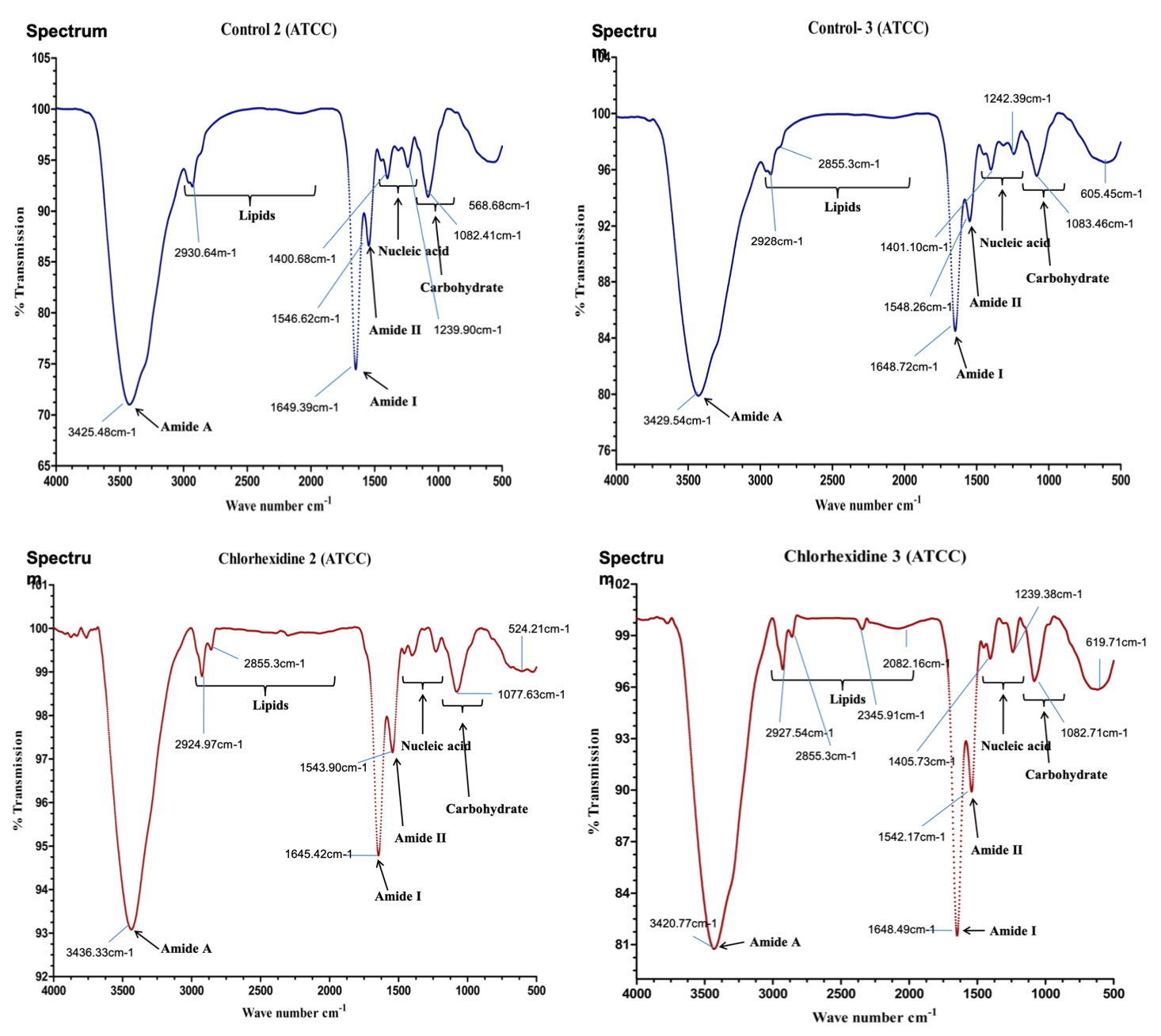

Supplement: S4 Fig — (TIFF) [file pone.0224107.s004.tiff]
